# Risk variants of the α-synuclein locus and REM sleep behavior disorder in Parkinson’s disease: a genetic association study

**DOI:** 10.1186/s12883-018-1023-6

**Published:** 2018-02-21

**Authors:** Kari Anne Bjørnarå, Lasse Pihlstrøm, Espen Dietrichs, Mathias Toft

**Affiliations:** 1 Department of Neurology, Drammen Hospital, Vestre Viken Hospital Trust, Dronninggata 21, 3004 Drammen, Norway; 2 Department of Neurology, Oslo University Hospital, Oslo, Norway; 3 Institute of Clinical Medicine, University of Oslo, Oslo, Norway

**Keywords:** *SNCA*, α-synuclein; genetics, REM sleep behavior disorder, RBD, Parkinson’s disease

## Abstract

**Background:**

Parkinson’s disease is a heterogeneous disorder where genetic factors may underlie clinical variability. Rapid eye movement sleep behavior disorder (RBD) is a parasomnia strongly linked to synucleinopathies, including Parkinson’s disease. We hypothesized that *SNCA* variants conferring risk of Parkinson’s disease would also predispose to an RBD phenotype.

**Methods:**

We assessed possible RBD (pRBD) status using the RBD screening questionnaire and investigated known susceptibility variants for Parkinson’s disease located in the α-synuclein (*SNCA*) and tau (*MAPT*) gene loci in 325 Parkinson’s disease patients. Associations between genetic risk variants and RBD were investigated by logistic regression, and an independent dataset of 382 patients from the Parkinson’s Progression Marker Initiative (PPMI) study was used for replication.

**Results:**

pRBD was associated with rs3756063 located in the 5’ region of *SNCA* (two-sided *p* = 0.018, odds ratio 1.44)*.* We replicated this finding in the PPMI dataset (one-sided *p* = 0.036, odds ratio 1.35) and meta-analyzed the results (two-sided *p* = 0.0032, odds ratio 1.40). The Parkinson’s disease risk variant in the 3′ region of *SNCA* and the *MAPT* variant showed no association with pRBD.

**Conclusions:**

Our findings provide proof of principle that a largely stable, dichotomous clinical feature of Parkinson’s disease can be linked to a specific genetic susceptibility profile. Indirectly, it also supports the hypothesis of RBD as relevant marker for a distinct subtype of the disorder.

## Background

Parkinson’s disease (PD) is a highly heterogeneous disorder. The prospect of linking clinical subtypes of PD to specific disease mechanisms and pathways would represent an important advance for optimization of clinical trials and, eventually, individualized treatment. Differences in the genetic basis of disease are often assumed to underlie variability in symptoms. Yet where large case-control studies have identified a range of common loci affecting disease risk for sporadic PD, the evidence for genetic modulation of the clinical presentation is currently scarce.

RBD is a parasomnia characterized by absence of muscle atonia during REM sleep. Affected individuals typically present vigorous motor activity and vocalization while they “live out their dreams”. Patients with idiopathic RBD are at high risk of developing neurodegenerative disease, in particular those disorders that are histopathologically characterized by α-synuclein aggregates [1]. The parasomnia is a frequent clinical feature in all synucleinopathies, including PD, where RBD affects about half of the patients and has been associated with more severe dysautonomia, cognitive impairment and increased risk of dementia [2, 3]. In contrast, RBD is rare in tauopathies like Alzheimer’s disease (AD) and progressive supranuclear palsy [4].

Synuclein and tau pathologies are neuropathological findings that despite considerable overlap reflect distinct disease processes. Both the α-synuclein (*SNCA)* and the tau (*MAPT)* gene regions are major susceptibility loci for sporadic PD, corroborated in several large GWAS [5]. Since RBD is strongly associated to synucleinopathies, we hypothesized that *SNCA* variants conferring risk of PD would also predispose to an RBD phenotype. Based on insights from neuropathology, we did not expect a similar effect of *MAPT* variability*.* We assessed RBD status and investigated SNPs representing *SNCA* and *MAPT* association signals in 325 PD patients from Norway, using data from PPMI as a replication set.

## Methods

### Subjects

A total of 325 patients diagnosed with PD by the UK Brain Bank criteria [6] were recruited from Oslo University Hospital and Drammen Hospital in South-East Norway. Patients of non-Scandinavian origin or with a known monogenic form of PD were excluded. The study was approved by the Regional Committee for Medical and Health Research Ethics in South-East Norway and all participants gave informed, written consent.

### Assessment of REM sleep behavior disorder

In our study, we used the RBD screening questionnaire (RBDSQ) to diagnose possible RBD (pRBD, i.e. history of dream enactment without polysomnographically confirmed REM sleep without atonia). RBDSQ is also included as part of the PPMI test battery. The RBDSQ consists of 13 yes/no questions, where six or more positive answers correspond to the optimal cut-off for detecting pRBD in PD. The questionnaire has been validated in the PD population with a sensitivity of 84% and a specificity of 96% [7]. Demographic characteristics and medication use of the included PD patient groups with and without pRBD are summarized in Table 1.

**Table 1:** Demographic characteristics and medication use

	Total (325)	pRBD (141)	Non-RBD (184)	*p* value
Male:female	213:112 (65.5)	106:35 (75.2)	107:77 (58.2)	< 0.01
Age, years	64.9 (9.4)	64.1 (9.4)	65.4 (9.4)	0.45
Disease duration, years	9.2 (6.9)	10.9 (7.2)	7.7 (6.4)	0.02
Levodopa yes:no	270:53 (83.6)	124:15 (87.9)	146:38 (79.3)	0.50
Agonist yes:no	213:110 (65.9)	80:59 (56.7)	133:51 (72.3)	0.03
SSRI/SNRI yes:no	52:269 (16.2)	32:106 (22.7)	20:163 (10.9)	0.02
Benzodiazepine yes:no	42:278 (15.1)	22:115 (15.6)	20:163 (10.9)	0.20
Clonazepam yes:no	19:302 (5.8)	12:126 (8.5)	7:176 (3.8)	0.50

Demographic data and medication use in PD patients with and without pRBD, respectively. Numbers in parentheses refers to percentage, except for age and disease duration, where it refers to standard deviation. *P* values refer to binary logistic regression analyses

Abbreviations: pRBD possible REM-sleep behavior disorder, *SSRI* selective serotonin reuptake inhibitor, *SNRI* serotonin and norepinephrine reuptake inhibitor

### Genotyping

We selected markers representing significant GWAS signals from our two loci of interest, including one SNP from *MAPT* (rs2942168) and three from *SNCA* (rs356165, rs3756063 and rs2245801). Across previous large-scale studies, the most significant *SNCA* variants have consistently been located near the 3’ end of the gene, tagged by rs356165 [8, 9]. However, in stepwise conditional analysis, an independent, secondary association signal has been identified in the 5’ region [10]. In studies with largely overlapping samples, different 5’ SNPs in only moderate linkage disequilibrium (LD) have been reported as secondary top-hits (rs2245801and rs7681154; r^2^ = 0.22, D’ = 0.99), and it is currently unclear whether these represent distinct or similar underlying functional variation [5, 8]. For reasons related to assay availability, rs7681154 was substituted by rs3756063 (r^2^ = 0.97) and rs1372520 was used as a proxy for rs2245801 (r^2^ = 0.88) in a subset of samples.

DNA was extracted from whole blood. Genotyping was performed using KASPar or TaqMan assays on a ViiA7 instrument (Life Technologies, Foster City, CA, USA). The genotyping rate was > 95% for all SNPs, and we observed no departure from Hardy-Weinberg equilibrium (*p* < 0.01).

### Replication in the PPMI dataset

Data on demographics, RBDSQ and genetic data genotyped on the NeuroX array were downloaded from the PPMI website. We identified 382 genotyped PD patients who had completed RBDSQ on at least one occasion and applied the same cutoff as in the discovery dataset to each patient’s median score. Genotype data was filtered by removing variants with minor allele frequency < 0.01, genotype call rate < 0.95 or Hardy-Weinberg equilibrium *p* > 1*10^− 6^, and imputed using the Michigan Imputation Server with data from the Haplotype Reference Consortuim as reference and otherwise default parameters [11], The SNP nominated as significant from the Norwegian dataset (rs3756063) was successfully imputed with a high quality score (r^2^ = 0.999). We further calculated principal components for the PPMI dataset based on a subset of common, linkage disequilibrium (LD)-pruned SNPs, using minor allele frequency > 0.05 and pairwise r^2^ < 0.5 as thresholds.

### Statistical analysis and power considerations

Statistical analyses were performed in PLINK [12] and SPSS v.21 (Armonk, New York, USA). Associations between SNP alleles and RBD status were assessed by logistic regression, including age and gender as covariates. For the replication analysis in PPMI data we also included the top four principal components in the regression model.

Notably, association studies of common symptoms within a disease group will typically have higher power compared to traditional case-control studies, due to the high prevalence of both outcomes. Based on 141 pRBD vs 184 non-RBD samples, we estimated the statistical power to detect an effect size of odds ratio 1.3 for a variant of allele frequency 0.5 to be 90% at *p* < 0.05, using the Genetic Power Calculator (zzz.bwh.harward.edu/gpc/cc2.html). Given the context of an exploratory study with a limited number of independent statistical tests, we set a threshold of *p* < 0.05 to nominate variants from the discovery analysis for further replication. For the one SNP assessed for replication in PPMI data, we hypothesized a consistent direction of effect, allowing for a one-sided *p*-value, and in the final meta-analysis, we set the significance threshold to *p* < 0.0125, adjusting for a total of 4 SNPs investigated in the study.

## Results

pRBD was more frequent in men (*p* < 0.01). The association results for SNPs versus pRBD status in PD patients, adjusted for age and gender by binary logistic regression, are shown in Table 2. pRBD was significantly associated with rs3756063 located in the 5′ region of *SNCA* (*p* = 0.018, odds ratio 1.44)*.* We observed no association for the other *SNCA* variants or *MAPT*. Repeating the same analysis in the independent PPMI dataset for rs3756063, we replicated the same result with marginal significance (one-sided *p* = 0.036, odds ratio 1.35), yet strikingly similar effect size and allele frequencies across groups. Meta-analysis of the results under a fixed effects model showed a two-sided *p*-value of 0.0032 and odds ratio 1.40. There was no indication of heterogeneity of effects across studies (heterogeneity index I^2^ = 0.00).

**Table 2:** Association results for pRBD with investigated *SNCA* and *MAPT* variants

dbSNP ID	Locus	Alleles	Frequency pRBD	Frequency Non-RBD	SE	OR	*p* value
Norwegian discovery dataset *N* = 141 pRBD-PD / 184 non-RBD-PD							
rs356165	*SNCA*	G/A	0.42	0.42	0.163	1.07	0.76
rs3756063	*SNCA*	C/G	0.59	0.48	0.155	1.44	0.018
rs2245801	*SNCA*	T/C	0.11	0.14	0.261	0.79	0.36
rs2942168	*MAPT*	T/C	0.14	0.11	0.239	1.27	0.33
PPMI replication dataset: *N* = 106 pRBD-PD / 276 non-RBD-PD							
rs3756063	*SNCA*	C/G	0.56	0.49	0.168	1.35	0.036^a^
Meta-analysis across both datasets for rs3756063:					0.114	1.40	0.0032

The association between SNPs and RBD was tested in a logistic regression model, including age and gender as covariates. The first allele listed is the effect allele

Abbreviations: *SNP* single-nucleotide polymorphism, *PD* Parkinson’s disease, *pRBD* possible REM-sleep behavior disorder, *OR* odds ratio, *SE* standard error

^a^One-sided p-value

## Discussion

RBD is highly prevalent in the PD population, and the present study is to our knowledge the first to explore genetic risk factors for pRBD within this context. The idiopathic form of the parasomnia is rare, yet intensively researched as one of the strongest known clinical markers of prodromal neurodegenerative disease. A recent case-control study investigated top-hits from PD GWAS in 261 patients with idiopathic RBD and 379 healthy controls, reporting significant associations with *MAPT* and *SCARB2* [13]. Assuming that *MAPT* could conceivably be an equally strong risk factor for PD without RBD, their findings are not necessarily in conflict with the lack of association with *MAPT* seen in the present study. Although other loci were negative, the authors noted non-significant odds ratios that resembled those seen in PD for several other signals, including *SNCA*. Importantly, the secondary *SNCA* GWAS signal highlighted in the present study was not investigated.

Knowledge about the contributions of common genetic variants to clinical variability in sporadic PD is currently limited. *SNCA* variability has been reported to be associated with both motor progression and cognitive outcomes, but results have varied across studies [14–16]. Evidence is currently accumulating both for other single loci and for the cumulative genetic risk burden with respect to their influence on motor and cognitive progression, although most published studies are moderate in sample size [15, 17–19]. Where motor symptoms and cognition are strongly associated with age and disease duration, RBD is arguably a more distinctly dichotomous clinical feature, showing weaker association to most demographic data. RBD occurs only in a proportion of PD patients and may appear even before motor symptoms [4]. RBD has been interpreted as a probable indicator of more extensive neuropathology in PD [2, 3]. Our finding suggests a specific genetic architecture predisposing to PD with pRBD, strengthening the rationale for RBD as marker for a dichotomy into PD subtypes.

The associated variant is located in the 5′ region of *SNCA* and represents a secondary GWAS signal, emerging as significant when conditioning on the 3′ top-hit SNP. While the causal mechanisms linking genetic variation to disease processes on a molecular scale are currently unknown, they are likely to involve complex regulatory networks specific to tissue subpopulations [20, 21]. Possibly, the discrepancy between the 3′ and 5′ variants’ effect on pRBD observed in the present study might reflect regional differences in neuronal *SNCA* regulation, indicating that multiple genetic risk variants at the same locus may carry a distinct pathogenic relevance.

We find that disease duration is longer in PD patients with pRBD. This is seen in some studies [22, 23]. However, several studies have shown that RBD can precede motor symptoms by decades, and thus the impact of disease duration on RBD symptoms is still unclear. Additionally, use of antidepressants is higher in the pRBD group. Antidepressants may increase the frequency of RBD, and could thus be a confounder [24].

A limitation of our study is the lack of polysomnography, as we are not able to identify RBD with absolute certainty. However, RBDSQ is widely used in larger studies, including the PPMI. Furthermore, according to the DSM-5 criteria for RBD, a suggestive history is enough to diagnose RBD in patients with an established synucleinopathy. Mean disease duration is 9 years, which supports the clinical diagnose of PD.

## Conclusion

pRBD was significantly associated with a known GWAS-hit variant in the 5′ region of *SNCA*, highlighting how distinctive clinical features in PD may depend on the genetic basis of disease.

Our study provides proof of principle for the integration of clinical, neuropathological and genetic observations to gain novel insights into the heterogeneous nature of sporadic PD. We anticipate a range of similar genotype-phenotype correlations emerging in future studies, mapping the clinicogenetic landscape of PD, with important implications for future therapeutic strategies.

## Abbreviations

GWAS: Genome-wide association study
PD: Parkinson’s disease
PPMI: Parkinson’s Progression Marker Initiative
pRBD: Possible REM sleep behavior disorder
RBD: REM sleep behavior disorder
RBDSQ: RBD Screening Questionnaire
REM: Rapid eye movement
SNP: Single-nucleotide polymorphism
